# ACEI/ARB Underused in Patients with Type 2 Diabetes in Chinese Population (CCMR-3B Study)

**DOI:** 10.1371/journal.pone.0116970

**Published:** 2015-02-12

**Authors:** Qionghong Xie, Chuan-Ming Hao, Linong Ji, Dayi Hu, Tongying Zhu, Xuehai Li, Dandan Qin, Danyi Zhang

**Affiliations:** 1 Division of Nephrology, Huashan Hospital, Fudan University, Shanghai, China; 2 Department of Endocrinology and Metabolism, Peking University People’s Hospital, Beijing, China; 3 Department of Cardiology, Peking University People’s Hospital, Beijing, China; 4 VitalStrategic Research Institute, Berwyn, PA, United States of America; Shanghai Institute of Hypertension, CHINA

## Abstract

**Objective:**

In patients with diabetic kidney disease, it is well documented that RAS blockade is associated with an improved outcome. This observational, multicenter study examined the “real-world” use of ACEI/ARB in patients with type 2 diabetes (T2DM) in China.

**Method:**

Data from the China Cardiometabolic Registries on blood pressure, blood lipid and blood glucose in Chinese T2DM patients (CCMR-3B) were used for the present study. Consecutive outpatients with T2DM for more than 6 months were recruited to this non-interventional, observational, cross-sectional study. Albuminuria was defined as urine albumin creatinine ratio (ACR) ≥ 30mg/g.

**Results:**

A total of 25,454 outpatients with T2DM from 6 regions in China were enrolled, 47.0% were male, and 59.8% had hypertension. ACR was measured in 6,383 of these patients and 3,231 of them ≥ 30mg/L. Among patients with hypertension, 73.0% were on antihypertensives, and 39.7% used ACEI/ARB. Of the 2,157 patients with hypertension and albuminuria, only 48.3% used ACEI/ARB. Among the non-hypertensive patients with albuminuria, ACEI/ARB usage was < 1%. Multivariate analysis revealed that comorbidities, region, hospital tier, physician specialty and patient’s educational level were associated with ACEI/ARB use.

**Conclusion:**

In T2DM with hypertension and albuminuria in China, more than half of them were not treated with ACEI/ARB. This real world evidence suggests that the current treatment for patients with diabetes coexisting with hypertension and albuminuria in China is sub-optimal.

## Introduction

Type 2 diabetes mellitus (T2DM) is a highly prevalent disease with a significant associated risk for cardiovascular morbidity and mortality [1–3]. According to the World Health Organization (WHO), as of 2010, an estimated 285 million people worldwide had diabetes, 90% of whom had T2DM. Its incidence is increasing rapidly worldwide, and by 2030, this number is projected to be 439 million [4–6]. In China, recent studies show that the prevalence of type 2 diabetes in adults is 9.7%-11.6% of the population, with an estimated 92–113.9 million individuals affected [7,8]. Importantly, the epidemic of diabetes and prediabetes in China has no sign of abating [7–9]. High prevalence of diabetes may also translate to a major epidemic of diabetes-related complications, including chronic kidney disease. The epidemic of diabetes and its related complications constitute not only a big threat to people’s health, but also a huge financial burden to patients and their families and society. Strategies to both prevent the development of and slow the progression of diabetes related complications would be of great importance for both patients and society. Since the patients with diabetes in China account for almost a half of the global prevalence, optimized management of diabetes in China will have a significant impact on the global burden of diabetes and its complications.

Strong evidences demonstrate that pharmacological blockade of the renin-angiotensin system (RAS) significantly improves the outcome of patients with diabetes. Angiotensin receptor blocker (ARB) significantly reduces the progression of micro-albuminuria to overt diabetic nephropathy in the patients with diabetes and hypertension [10]. ARBs also show strong renal protection in patients with overt diabetic nephropathy, significantly slowing the decline of renal function in these patients [11,12]. Sub-analysis shows that the Asian population responds better to ARB therapy in protecting the kidney from end-stage renal disease (ESRD) when compared to the Black and Hispanic [13]. The beneficial effect of RAS blockade on the diabetic kidney is attributable to its direct renal effect, in addition to its blood pressure lowing effect. In contrast, calcium channel blocker (CCB) failed to show reno-protection when compared to ARB in this population [14]. Compelling evidence also showed that ACEI/ARB is associated with reduced cardiovascular morbidity and mortality in the patients with diabetes, hypertension and/or albuminuria [15]. Based on these strong evidences, guidelines from American Diabetes Association (ADA) and Kidney Disease Improving Global Outcomes (KDIGO) recommend either ACE inhibitors or ARBs being used in the treatment of diabetic patients with micro- or macro-albuminuria [16,17].

The aim of this study was to examine how well the above evidences were reflected in our real world clinical practice in China, using data from CCMR-3B, a nationally representative sample of the diabetic population in China [18].

## Materials and Methods

### Patients

The adult outpatients who had been diagnosed as type 2 diabetes mellitus for more than six months according to the WHO criteria, as recommended by the Chinese diabetes guidelines were recruited to this non-interventional, observational, cross-sectional study between August 2010 and March 2011 [19]. All the patients needed to have medical cross-sectional study between August 2010 and March 2011 [19]. All the patients needed to have medical records or could present their disease history. The patients with type 1 diabetes, and who were pregnant, or participating in other clinical study were not included. Consecutive outpatients were from 104 hospitals in six regions including the Northeast (Liaoning Province), North (Beijing), East (Shanghai), Northwest (Shaanxi and Gansu province), Southwest (Sichuan province and Chongqing) and Central south (Guangdong and Hunan Province) [18]. This study was approved by the Medical Ethics Committee of Peking University People’s Hospital and all of the patients provided their written informed consent.

### Clinical data collecting

For enrolled patients, self-reported information on demographics, socio-economic status (i.e. level of education, employment status), medical history (including family history of diabetes and cardiovascular diseases), co-morbidities, and concurrent medications were collected. Patients were asked whether they were aware of having been previously diagnosed with hypertension or dyslipidemia, and whether they were receiving blood pressure lowering drugs prior to their enrollment. In addition, the patients’ visiting hospital tier and department were also recorded. Pre-specified clinical and laboratory data, including HbA_1_C, serum glucose, serum lipid profile (total cholesterol, low-density lipoprotein, high-density lipoprotein, triglyceride), serum creatinine, urine creatinine, urine micro-albumin/albumin, and albumin to creatinine ratio (ACR) were collected. Laboratory measurements were obtained within 30 days before or 7 days after screening [18]. Albuminuria was defined as micro-albuminuria (urinary albumin creatinine ratio (ACR) 30–300 mg/g) or macro-albuminuria (ACR ≥ 300 mg/g) in two out of three consecutive samples within 6 months. eGFR was calculated using the modified four-variable Modification of Diet in Renal Disease (MDRD) study equation [20].

### Statistical analysis

Demographic and clinical information was recorded at baseline. Continuous variables are presented as mean ± SD (normal distribution) or median with range of quartile (non-normal distribution), and categorical variables are expressed as frequencies and percentages. Comparisons between groups were analyzed using independent t test or Mann-Whitney U test for continuous variables, and Pearson chi-squared test or Fisher exact test for categorical variables. In a multivariate analysis, binary logistic regression was used to identify independent associated factors of ACEI/ARB use in whole population and hypertension population. The criterion for significance was P<0.05.

## Results

### Patient characteristics

A total of 25454 adult outpatients with type 2 diabetes were enrolled into this study. The median age of the study population was 63 with quartile range 55 to 72 yrs. Forty-seven percent of them were male, 59.8% with hypertension and 44.8% with obesity (BMI ≥ 25 kg/m^2^). The median (quartile) level of HbA_1_C was 7.10% (6.25%-8.60%). Serum creatinine data were available in 22628 of these patients, and 20370 (90.0%) had an eGFR ≥ 60ml/min. Of the 25454 patients, 6383 (25.1%) had ACR measurements. Among these patients with ACR available, 2163 (33.9%) exhibited 30≤ACR<300mg/g and 1068 (16.7%) exhibited ACR≥300mg/g. (Table 1)

**Table 1:** Comparison of patient characteristics between hypertension and non-hypertension population.

	Total	HTN	non-HTN	p value
Number	25454	15234 (59.8%)	10220 (40.2%)	-
Age	63 (55–72)	66 (58–74)	58 (50–67)	<0.001
Male	11950 (47.0%)	6773 (44.5%)	5177 (50.7%)	<0.001
HbA_1_C (%)	7.10 (6.25–8.60)	7.10 (6.30–8.40)	7.20 (6.20–8.90)	<0.001
TG (mmol/L)	1.56 (1.10–2.27)	1.60 (1.12–2.30)	1.50 (1.05–2.22)	<0.001
TC (mmol/L)	4.88 (4.15–5.67)	4.87 (4.11–5.67)	4.90 (4.20–5.67)	0.005
BMI (kg/m^2^)	24.6 (22.5–26.8)	25.0 (22.9–27.3)	24.1 (22.0–26.2)	<0.001
Albuminuria				<0.001
Normal	3152 (12.4%)	1565 (10.3%)	1587 (15.5%)	
Micro-albuminuria	2163 (8.5%)	1373 (9.0%)	790 (7.7%)	
Macro-albuminuria	1068 (4.2%)	784 (5.2%)	284 (2.8%)	
N/A	19071 (74.9%)	11512 (75.6%)	7559 (74.0%)	
eGFR (ml/min*1.73m^2^)	107 (81–135)	102 (75–129)	116 (91–143)	<0.001
≥90	15544 (61.1%)	8541 (56.1%)	7003 (68.5%)	
90–60	4826 (19.0%)	3160 (20.7%)	1666 (16.3%)	
60–30	1563 (6.1%)	1182 (7.8%)	381 (3.7%)	
30–15	323 (1.3%)	286 (1.9%)	37 (0.4%)	
<15	372 (1.5%)	349 (2.3%)	23 (0.2%)	
N/A	2826 (11.1%)	1716 (11.3%)	1110 (10.9%)	
Antihypertensives usage rate	11182 (43.9%)	11120 (73.0%)	62 (0.61%)	<0.001
ACEI/ARB usage rate	6063 (23.8%)	6043 (39.7%)	20 (0.20%)	<0.001
CCB usage rate	6199 (24.4%)	6184 (40.6%)	15 (0.15%)	<0.001

Abbreviation: HTN, hypertension; HbA_1_C, glycated hemoglobin; TG, total glyceride; TC, total cholesterol; BMI, body mass index; eGFR, estimated glomerular filtration rate; ACEI, angiotensin converting enzyme inhibitor; ARB, angiotensin receptor blocker; CCB, calcium channel blocker.

Compared with the non-hypertensive patients, hypertensive patients were older, more often women, and had lower HbA_1_C and total cholesterol levels, but had higher total glyceride and serum creatinine. The rate of ACR measurements in hypertensive patients was lower than in the non-hypertensive patients (24.4% vs. 26%, p = 0.004). In non-hypertensive patients, the usage of antihypertensive medicines ACEI/ARB was lower than 1%, significantly lower than in the hypertensive patients (p<0.0001). These results showed that hypertension was the main determinant of the ACEI/ARB application, so further analysis was focused on the hypertension sub-group. (Table 1)

### Antihypertensive prescriptions in Chinese 2-DM patients with hypertension

Of the 15234 patients with hypertension, 3722 (24.4%) had ACR measurements. Compared with hypertensive patients without ACR measurements, hypertensive patients with ACR measurements were younger, and had higher HbA_1_C, higher total cholesterol and total glyceride (Table 2). Multivariate analysis showed that the detection of ACR was associated with age, region, visiting hospital tier and department (p<0.01 for all above). eGFR was not significantly associated with the ACR detection.

**Table 2:** Comparison of characteristics between subjects with and without ACR measurements in patients with hypertension.

	HTN	Measurement	No measurement	P value
Number	15234	3722 (24.4%)	11512 (75.6%)	-
Age	66 (58–74)	65 (57–73)	66 (58–74)	<0.001
Male	6773 (44.5%)	1637 (44.0%)	5136 (44.6%)	0.500
HbA_1_C	7.10 (6.30–8.40)	7.20 (6.30–8.70)	7.06 (6.20–8.30)	<0.001
TG (mmol/L)	1.60 (1.12–2.30)	1.64 (1.15–2.48)	1.58 (1.11–2.26)	<0.001
TC (mmol/L)	4.87 (4.11–5.67)	4.96 (4.21–5.80)	4.83 (4.09–5.62)	<0.001
BMI	25.0 (22.9–27.3)	25.0 (22.9–27.3)	25.0 (22.9–27.3)	0.584
eGFR (ml/min*1.73m^2^)	102 (75–130)	102 (78–130)	101 (75–129)	0.073
Hospital tier				<0.001
1st tier	3885 (25.5%)	545 (14.6%)	3340 (29.0%)	
2nd tier	5688 (37.3%)	1570 (42.2%)	4118 (35.8%)	
3rd tier	5661 (37.2%)	1607 (43.2%)	4.54 (35.2%)	
Region				<0.001
East	2872 (18.9%)	815 (21.9%)	2057 (17.9%)	
North	3018 (19.8%)	332 (8.9%)	2686 (23.3%)	
Southwest	2764 (18.1%)	949 (25.5%)	1815 (15.8%)	
Northeast	2601 (17.1%)	765 (20.6%)	1836 (15.9%)	
Central south	2099 (13.8%)	682 (18.3%)	1417 (12.3%)	
Northwest	1880 (12.3%)	179 (4.8%)	1701 (14.8%)	
Department				<0.001
Cardiology	2781 (18.3%)	580 (15.6%)	2201 (19.1%)	
Nephrology	1155 (7.6%)	314 (8.4%)	841 (7.3%)	
Endocrinology	6253 (41.0%)	1984 (53.3%)	4269 (37.1%)	
Internal Medicine/Others	5.45 (33.1%)	844 (22.7%)	4201 (36.5%)	

Abbreviation: HTN, hypertension; HbA_1_C, glycated hemoglobin; TG, total glyceride; TC, total cholesterol; BMI, body mass index; eGFR, estimated glomerular filtration rate.

In the hypertensive patients, 73.0% were on anti-hypertensive medications, and 39.7% used ACEI/ARB, 40.6% used CCB. (Table 3) In the patients with hypertension and albuminuria (n = 2157), 78.9% were on anti-hypertensive medications, 48.3% were on ACEI/ARB (n = 1041). Of those who were treated with non-ACEI/ARB antihypertensive medications (n = 661), 74.5% (n = 493) were treated with CCBs. (Table 4) Furthermore, of the patients with hypertension and macro-albuminuria (N = 784), still only 55.1% were treated with ACEI/ARB. Among the hypertensive patients without ACR detection, the usage rate of ACEI/ARB was 38%, significantly lower than that in hypertensive patients with (48.3%) or without (42.4%) albuminuria.

**Table 3:** The usage rates of antihypertensive medications, ACEI/ARB and CCB in patients with hypertension.

eGFR (ml/min·1.73m^2^)	HTN	urine albumin		
		positive	negative	N/A
Number				
Total	15234	2157 (14.2%)	1565 (10.3%)	11512 (75.5%)
≥90	8541 (56.1%)	1241 (57.5%)	1142 (73.0%)	6158 (53.5%)
90–60	3160 (20.7%)	510 (23.7%)	319 (20.4%)	2331 (20.2%)
60–30	1182 (7.7%)	244 (11.3%)	69 (4.4%)	869 (7.6%)
30–15	286 (1.9%)	62 (2.9%)	5 (0.3%)	219 (1.9%)
<15	349 (2.3%)	57 (2.6%)	3 (0.2%)	289 (2.5%)
N/A	1716 (11.3%)	43 (2.0%)	27 (1.7%)	1646 (14.3%)
Antihypertensives usage rate				
Total	11120 (73.0%)	1702 (78.9%)	1193 (76.2%)	8225 (71.4%)
≥90	6207 (72.7%)	951 (76.6%)	854 (74.8%)	4402 (71.5%)
90–60	2344 (74.2%)	422 (82.7%)	259 (81.2%)	1663 (71.3%)
60–30	934 (79.0%)	204 (83.6%)	57 (82.6%)	673 (77.4%)
30–15	231 (80.8%)	49 (79.0%)	4 (80.0%)	178 (81.3%)
<15	268 (76.8%)	45 (78.9%)	1 (33.3%)	222 (76.8%)
N/A	1136 (66.2%)	31 (72.1%)	18 (66.7%)	1087 (66.0%)
ACEI/ARB usage rate				
Total	6043 (39.7%)	1041 (48.3%)	663 (42.4%)	4339 (38.0%)
≥90	3496 (40.9%)	559 (45.0%)	482 (42.2%)	2455 (39.9%)
90–60	1306 (41.3%)	273 (53.5%)	145 (45.5%)	888 (38.1%)
60–30	550 (46.5%)	133 (54.5%)	27 (39.1%)	390 (44.9%)
30–15	103 (36.0%)	30 (48.4%)	1 (20.0%)	72 (32.9%)
<15	129 (37.0%)	25 (43.9%)	0	104 (36.0%)
N/A	459 (26.8%)	21 (48.8%)	8 (29.6%)	430 (26.1%)
CCB usage rate				
Total	6184 (40.6%)	961 (44.6%)	650 (41.5%)	4574 (39.7%)
≥90	3261 (38.2%)	514 (41.4%)	453 (39.7%)	2294 (37.3%)
90–60	1327 (42.0%)	243 (47.7%)	150 (47.0%)	934 (40.1%)
60–30	579 (49.0%)	121 (49.6%)	36 (52.2%)	422 (48.6%)
30–15	169 (59.1%)	32 (51.6%)	3 (60.0%)	134 (61.2%)
<15	213 (61.0%)	39 (68.4%)	1 (33.3%)	173 (59.9%)
N/A	635 (37.0%)	11 (25.6%)	7 (25.9%)	617 (37.5%)

Abbreviation: HTN, hypertension; N/A, no available; ACEI, angiotensin converting enzyme inhibitor; ARB, angiotensin receptor blocker; CCB, calcium channel blocker; eGFR, estimated glomerular filtration rate.

**Table 4:** Further analysis of antihypertensive regimens in patients with type 2 diabetes, hypertension and albuminuria (n = 2157).

eGFR (ml/min·1.73m^2^)	ACEI/ARB usage		Non-ACEI/ARB usage			Total
	Single drug	Combination	CCB used	Other used	Unused	
Total	484 (22.5%)	557 (25.8%)	493 (22.9%)	168 (7.7%)	455 (21.1%)	2157
≥90	283 (22.0%)	286 (23.0%)	274 (22.1%)	118 (9.5%)	290 (23.4%)	1241
90–60	127 (24.9%)	146 (28.6%)	121 (23.7%)	28 (5.5%)	88 (17.2%)	510
60–30	53 (21.7%)	80 (32.8%)	54 (22.1%)	17 (7.0%)	40 (16.4%)	244
30–15	13 (21.0%)	17 (27.4%)	19 (30.6%)	0	13 (21.0%)	62
<15	4 (7.1%)	21 (36.8%)	19 (31.7%)	1 (1.8%)	12 (21.1%)	57
N/A	14 (32.5%)	7 (16.3%)	6 (14.0%)	4 (9.3%)	12 (27.9%)	43

Abbreviation: eGFR, estimated glomerular filtration rate; ACEI, angiotensin converting enzyme inhibitor; ARB, angiotensin receptor blocker; CCB, calcium channel blocker; N/A, no available.

To determine whether a low rate of ACEI/ARB use is associated with renal function, we examined their uses in different eGFR levels. As shown in Table 3, in patients with hypertension and albuminuria, 92.5% were in CKD (chronic kidney disease) stages 1, 2 and 3. The ACEI/ARB usage rates of those three stages were 45%, 53.5% and 54.5% respectively. These data suggested that the low ACEI/ARB usage rate in this population was not due to the advanced stages of CKD.

### The determinants of ACEI/ARB use in hypertensive patients

**Fig 1:**
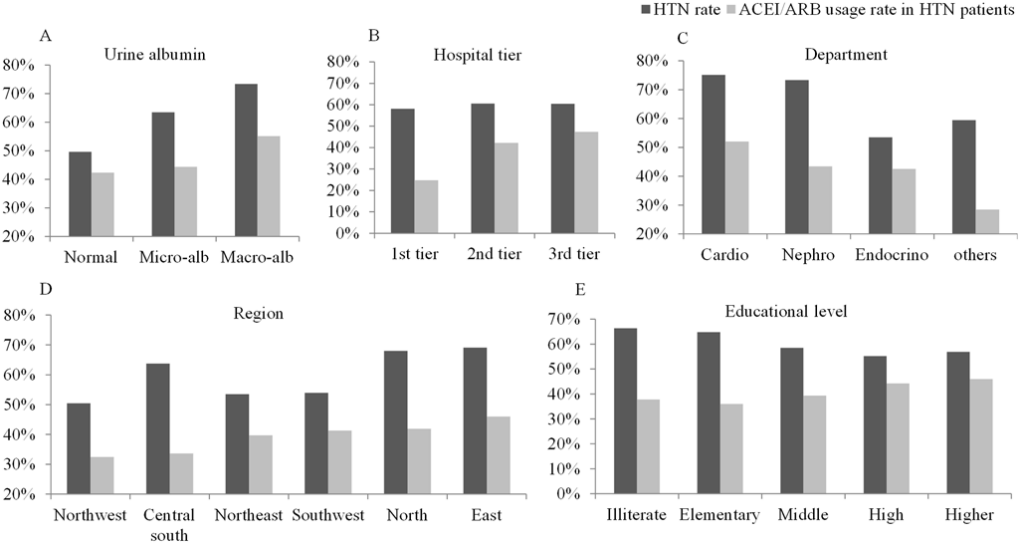
Hypertension rate of the study population and ACEI/ARB usage rate in the hypertensive patients. Patients with higher ACR and lower educational level had higher rate of hypertension (p < 0.001 for both); Compared with cardiology and nephrology departments, patients visited in endocrinology department had lower rate of hypertension (p < 0.001); There were not significant differences in region and visiting hospital titer (p > 0.05 for both). In hypertension population, ACEI/ARB usage rate was higher in patients with higher ACR (p < 0.0001), higher educational level (p < 0.0001) and higher tier of visiting hospital (p < 0.0001); Cardiologists had the highest usage rate, while internal medicine/others had the lowest (p < 0.0001); East China had the highest usage rate, with the North, Southwest and Northeast regions following, while Central, South and Northwest China had relatively lower rates (p < 0.0001).

As listed in Table 5, male gender, higher education level, hospital tier and ACR had a higher usage rate of ACEI/ARB in patients with Type 2 diabetes and hypertension. Region and physician specialty were also associated with the usage rates. East China had the highest usage rate, with the North, Southwest and Northeast regions following, while Central, South and Northwest China had relatively lower rates. Cardiologists had the highest usage rate, while internal medicine/others had the lowest. (Fig. 1) There were no significant differences in ACEI/ARB usage in different occupations and eGFR. A multivariate analysis revealed that age, BMI, hypertension, ACR, hospital tier, region, physician specialty and education level were independently associated with the ACEI/ARB usage rate in the study of the whole population. Similarly, age, BMI, ACR, hospital tier, region, physician specialty and education level were also independent determents of ACEI/ARB usage in the hypertensive patients. (Table 6)

**Table 5:** Univariate analysis for associated factors of ACEI/ARB usage in patients with hypertension.

	HTN	ACEI/ARB used	ACEI/ARB unused	p value
Number	15234	6043 (39.7%)	9191 (60.3%)	-
Age	66 (58–74)	66 (58–74)	66 (58–74)	0.161
Male	6773 (44.5%)	2807 (41.4%)	3966 (58.6%)	<0.001
Albuminuria				<0.001
Normal	1565 (10.3%)	663 (11.0%)	902 (9.8%)	
Micro-albuminuria	1373 (9.0%)	609 (10.1%)	764 (8.3%)	
Macro-albuminuria	784 (5.1%)	432 (7.1%)	352 (3.8%)	
N/A	11512 (75.6%)	4339 (71.8%)	7173 (78.1%)	
Hospital tier				<0.001
1st tier	3885 (25.5%)	961 (15.9%)	2924 (31.8%)	
2nd tier	5688 (37.3%)	2402 (39.7%)	3286 (35.8%)	
3rd tier	5661 (37.2%)	2680 (44.4%)	2981 (32.4%)	
Region				<0.001
East	2872 (18.9%)	1320 (21.8%)	1552 (16.9%)	
North	3018 (19.8%)	1264 (20.9%)	1754 (19.1%)	
Southwest	2764 (18.1%)	1142 (18.9%)	1622 (17.6%)	
Northeast	2099 (13.8%)	833 (13.8%)	1266 (13.8%)	
Central south	2601 (17.1%)	874 (14.5%)	1727 (18.8%)	
Northwest	1880 (12.3%)	610 (10.1%)	1270 (13.8%)	
Department				<0.001
Cardiology	2781 (18.3%)	1447 (24.0%)	1334 (14.5%)	
Nephrology	1155 (7.6%)	502 (8.3%)	653 (7.1%)	
Endocrinology	6253 (41.0%)	2660 (44.0%)	3593 (39.1%)	
Internal Medicine/Others	5045 (33.1%)	1434 (23.7%)	3611 (39.3%)	
Education level				<0.001
Illiterate	1125 (7.4%)	425 (7.0%)	700 (7.6%)	
Elementary school	3672 (24.1%)	1322 (21.9%)	2350 (25.6%)	
Middle school	6987 (45.9%)	2749 (45.5%)	4238 (46.1%)	
High school	2175 (14.3%)	961 (15.9%)	1214 (13.2%)	
Higher	1275 (8.4%)	586 (9.7%)	689 (7.5%)	
Ocupation				0.088
Unemployed	1656 (10.9%)	591 (9.8%)	1065 (11.6%)	
Full-time	1742 (11.4%)	737 (12.2%)	1005 (10.9%)	
Part-time	377 (2.5%)	146 (2.4%)	231 (2.5%)	
Retired	11459 (75.2%)	4569 (75.6%)	6890 (75.0%)	
HbA_1_C	7.10 (6.30–8.40)	7.10 (6.30–8.40)	7.10 (6.20–8.40)	0.002
TG (mmol/L)	1.60 (1.12–2.30)	1.56 (1.10–2.27)	1.61 (1.14–2.33)	<0.001
TC (mmol/L)	4.87 (4.11–5.67)	4.77 (4.00–5.56)	4.94 (4.18–5.74)	<0.001
BMI (kg/m^2^)	25.0 (22.9–27.3)	25.1 (23.0–27.4)	24.9 (22.8–27.2)	<0.001
eGFR (ml/min·1.73m^2^)	102 (75–129)	101 (75–128)	102 (76–130)	0.187

Abbreviation: ACEI, angiotensin converting enzyme inhibitor; ARB, angiotensin receptor blocker; HTN, hypertension; HbA_1_C, glycated hemoglobin; TG, total glyceride; TC, total cholesterol; BMI, body mass index; eGFR, estimated glomerular filtration rate; N/A, no available.

**Table 6:** Multivariate analysis for associated factors of ACEI/ARB usage in total population and hypertension population.

	Total population		HTN patients	
	OR	p value	OR	p value
Age	1.006 (1.003–1.009)	0.0005	1.006 (1.002–1.009)	0.0011
Male	1.038 (0.967–1.114)	0.3405	1.035 (0.964–1.111)	0.3424
HTN	326.4 (210.0–507.3)	<0.0001	-	-
Albuminuria		<0.0001		<0.0001
Normal	1		1	
Micro-albuminuria	1.120 (0.964–1.300)		1.114 (0.958–1.294)	
Macro-albuminuria	1.357 (1.134–1.624)		1.339 (1.118–1.603)	
N/A	0.885 (0.792–0.989)		0.875 (0.783–0.978)	
Hospital tier		<0.0001		<0.0001
1st tier	1		1	
2nd tier	1.901 (1.678–2.154)		1.887 (1.665–2.139)	
3rd tier	2.167 (1.866–2.517)		2.154 (1.854–2.503)	
Region		<0.0001		<0.0001
East	1		1	
North	0.725 (0.648–0.810)		0.726 (0.649–0.811)	
Central South	0.784 (0.702–0.875)		0.775 (0.694–0.866)	
South	0.790 (0.693–0.900)		0.794 (0.696–0.905)	
Northwest	0.511 (0.455–0.575)		0.511 (0.455–0.575)	
West	0.564 (0.494–0.640)		0.565 (0.496–0.643)	
Department		<0.0001		<0.0001
Cardiology	1		1	
Nephrology	0.653 (0.594–0.718)		0.661 (0.601–0.727)	
Endocrinology	0.591 (0.513–0.682)		0.601 (0.521–0.693)	
Internal Medicine/Others	0.588 (0.509–0.679)		0.593 (0.514–0.685)	
Education		<0.0001		<0.0001
Illiterate	1		1	
Elementary school	0.924 (0.801–1.066)		0.925 (0.801–1.067)	
Middle school	1.127 (0.981–1.294)		1.130 (0.983–1.298)	
High school	1.331 (1.134–1.560)		1.338 (1.141–1.570)	
Higher	1.465 (1.228–1.748)		1.469 (1.231–1.753)	
HbA_1_C	1.006 (0.988–1.025)	0.5045	1.005 (0.987–1.024)	0.5604
TG (mmol/L)	0.988 (0.973–1.004)	0.1455	0.988 (0.972–1.003)	0.1238
TC (mmol/L)	0.991 (0.972–1.010)	0.3475	0.991 (0.972–1.010)	0.3631
BMI (kg/m^2^)	1.023 (1.014–1.033)	<0.0001	1.023 (1.013–1.032)	<0.0001

Abbreviation: HTN, hypertension; N/A, no available; HbA_1_C, glycated hemoglobin; TG, total glyceride; TC, total cholesterol; BMI, body mass index.

## Discussion

The aim of this study was to assess the utilization of ACEI/ARB in patients with type 2 diabetes, whether with albuminuria or not in China. The results showed that in patients with diabetes coexisting with hypertension and albuminuria, 48.3% used ACEI/ARB as antihypertensive drugs, 22.9% used CCB without ACEI/ARB and 21.1% were not on any antihypertensive medications; in patients with diabetes, hypertension and macro-albuminuria, only 55.1% were on ACEI/ARB. Less than 1% patients with diabetes and albuminuia but without hypertension used ACEI/ARB. These results suggested that the treatments of diabetic patients with hypertension and kidney damage are not optimized in the Chinese Type 2 diabetic population. Factors that influenced ACEI/ARB use include comorbidities, region, physician specialty, hospital tier, and education levels of the patients.

It is well established that ACEI/ARB is renal protective in patients with diabetes and kidney damage [10–12]. Mounting evidences suggest that ACEI/ARB is also associated with reduced cardiovascular morbidity and mortality in patients with diabetes and hypertension regardless of albuminuria [21]. Furthermore, early use of ARB in patients with hypertension and Type 2 diabetes has been demonstrated to be cost-effective with more overall savings in health care resource utilization [22,23]. However, in the present real world survey, ACEI/ARBs were used only in less than 50% of patients with diabetes, hypertension and albuminuria, and in less than 1% of patients with diabetes and albuminuria but without hypertension. This low rate of ACEI/ARB use does not appear to be associated with advanced renal function damage because 92.5% of patients were in CKD stages 1 through 3, and their ACEI/ARB usage rate is 45%, 53.5% and 54.5% in CKD stages 1, 2 and 3 respectively. In the United States, Yang reported 63% of patients with diabetes, hypertension and renal disease, 58.3% of patients with diabetes and hypertension but without renal disease and 43.1% of patients with diabetes and renal involvement but without hypertension were administered ACEI/ARB [24]. Rosen et al reported that 54% of patients with albuminuria, 64% of patients with hypertension, and 74% of patients with both conditions were prescribed ACEI/ARB in 2000 [25]. A study from Taiwan also showed that over 50% of patients in the CKD at-risk group (defined as patients with diabetes and/or hypertension but no proteinuria) and in CKD stages 1–5 were prescribed ACEI/ARB [26]. From these studies, it seems that the managements of diabetes with kidney damage in these areas are sub-optimized in some patients based on current evidences. Improved diabetic management in this population may have an important impact on the burden of diabetes-related complications.

In our study, the usage of ACEI/ARB was associated with albuminuria (Table 6). This association only existed in patients with macro-albuminuria, but not in patients with micro-albuminuria. This may suggest that micro-albuminuria had not drawn enough attention from the physician as well as patients. Besides, in the non-hypertensive patients regardless of albuminuria, the usage of ACEI/ARB was less than 1%, which was significantly lower than previous reports in other countries [27]. ARBs have been shown to reduce the rate of progression from micro- to macroalbuminuria as well as ESRD in patients with Type 2 diabetes regardless of hypertension [10–12,16]. Thus, the utilization of ACEI/ARB in the patients with diabetes and albuminuria should be given more attention.

The present study showed that patients visiting cardiologists were more likely to receive ACEI/ARB treatment than when visiting other departments. This may be associated with comorbidities such as coronary artery disease or congestive heart failure, which represent separate indications for ACEI/ARB. In the previous study, greater rates of ACEI/ARB use were found in patients with coronary artery disease or congestive heart failure [28]. We also found that the patients with higher BMI were more likely prescribed ACEI/ARB than their counterparts. This may be also associated with higher comorbidities of cardiovascular diseases in obese patients. The patients with higher HbA_1_C and lower total cholesterol/glyceride were also more likely prescribed ACEI/ARB, but this association was no longer significant in multivariate analysis.

The usage rates of ACEI/ARB in patients from West China and Northwest China, which are relatively under-developed, were significantly lower than those from the developed areas. The usage rate in patients visiting 1^st^ tier hospitals was only half of the rate in patients visiting 3^rd^ tier hospitals. This may suggest that more educational efforts should be made from the physicians of the developing areas and 1^st^ tier hospitals. Besides, patients visiting in the departments of general internal medicine other than specialties such as cardiology, endocrinology and nephrology also had significantly lower usage rates. These results suggest that not only is education important, but early referral to a specialist is as well.

In addition, the present study also showed that the control of hyperglycemia and dyslipidemia in this population is sub-optimized, with 55.1% with HbA_1_C > 7%, 63.8% with serum total cholesterol > 4.5mmol/L and 58.7% with BMI > 24kg/m^2^ (the cut-off value was set by reference to the Chinese guidelines for diabetes prevention and treatment) [29]. Adequate control of glycemia and dyslipidemia has been demonstrated to be beneficial to the patients with diabetes related complications [2,30,31].

There were several limitations in our study. First, only one-fourth of the patients had ACR detection. Further analysis showed that ACR measurement was not associated with eGFR. Whether the patients had ACR was associated with their region, visiting hospital tier and department. The patients with ACR detection had lower lower rate of hypertension, higher levels of HbA_1_C, total cholesterol and glyceride. However, the multivariate analysis also revealed that the usage of ACEI/ARB were not associated with HbA_1_C and total cholesterol/glyceride. Importantly, the usage of ACEI/ARB in the hypertensive patients without ACR (38%) were significantly lower than in patients with (48%) or without (42%) albuminuria, so the real usage of ACEI/ARB could be even lower in this population. Second, the diagnosis of hypertension was based on the patients’ self-report. This may overrate the ACEI/ARB usage in hypertensive patients but underrate the usage in non-hypertensive patients. Third, this was a hospital-based investigation and the usage of ACEI/ARB may be amplified in this population because patients who are not aware of their diseases may not go to the hospital.

### Conclusions

This study showed that the prescription of ACEI/ARB was not optimal in patients with Type 2 diabetes coexisting with hypertension and/or albuminuria in China. Comorbidities, region, hospital tier, physician specialty and education level were the independent impact factors of the ACEI/ARB usage. This study suggests that more educational efforts should be made to the physicians, especially those from relatively under-developed regions, lower tier hospitals.

## References

[1] Wilson PW (1998) Diabetes mellitus and coronary heart disease. Am J Kidney Dis 32: S89–100. 9820468 10.1053/ajkd.1998.v32.pm9820468

[2] Stratton IM, Adler AI, Neil HA, Matthews DR, Manley SE, et al (2000) Association of glycaemia with macrovascular and microvascular complications of type 2 diabetes (UKPDS 35): prospective observational study. BMJ 321: 405–412. 10938048 10.1136/bmj.321.7258.405 PMC27454

[3] Juutilainen A, Lehto S, Ronnemaa T, Pyorala K, Laakso M (2005) Type 2 diabetes as a “coronary heart disease equivalent”: an 18-year prospective population-based study in Finnish subjects. Diabetes Care 28: 2901–2907. 16306552 10.2337/diacare.28.12.2901

[4] Wild S, Roglic G, Green A, Sicree R, King H (2004) Global prevalence of diabetes: estimates for the year 2000 and projections for 2030. Diabetes Care 27: 1047–1053. 15111519 10.2337/diacare.27.5.1047

[5] Shaw JE, Sicree RA, Zimmet PZ (2010) Global estimates of the prevalence of diabetes for 2010 and 2030. Diabetes Res Clin Pract 87: 4–14. 10.1016/j.diabres.2009.10.007 19896746

[6] Chen L, Magliano DJ, Zimmet PZ (2012) The worldwide epidemiology of type 2 diabetes mellitus—present and future perspectives. Nat Rev Endocrinol 8: 228–236. 10.1038/nrendo.2011.183 22064493

[7] Yang SH, Dou KF, Song WJ (2010) Prevalence of diabetes among men and women in China. N Engl J Med 362: 2425–2426; 10.1056/NEJMc1004671 20578276

[8] Xu Y, Wang L, He J, Bi Y, Li M, et al (2013) Prevalence and control of diabetes in Chinese adults. JAMA 310: 948–959. 10.1001/jama.2013.168118 24002281

[9] Chan JC, Malik V, Jia W, Kadowaki T, Yajnik CS, et al (2009) Diabetes in Asia: epidemiology, risk factors, and pathophysiology. JAMA 301: 2129–2140. 10.1001/jama.2009.726 19470990

[10] Parving HH, Lehnert H, Brochner-Mortensen J, Gomis R, Andersen S, et al (2001) The effect of irbesartan on the development of diabetic nephropathy in patients with type 2 diabetes. N Engl J Med 345: 870–878. 11565519 10.1056/NEJMoa011489

[11] Lewis EJ, Hunsicker LG, Clarke WR, Berl T, Pohl MA, et al (2001) Renoprotective effect of the angiotensin-receptor antagonist irbesartan in patients with nephropathy due to type 2 diabetes. N Engl J Med 345: 851–860. 11565517 10.1056/NEJMoa011303

[12] Brenner BM, Cooper ME, de Zeeuw D, Keane WF, Mitch WE, et al (2001) Effects of losartan on renal and cardiovascular outcomes in patients with type 2 diabetes and nephropathy. N Engl J Med 345: 861–869. 11565518 10.1056/NEJMoa011161

[13] Chan JC, Wat NM, So WY, Lam KS, Chua CT, et al (2004) Renin angiotensin aldosterone system blockade and renal disease in patients with type 2 diabetes. An Asian perspective from the RENAAL Study. Diabetes Care 27: 874–879. 15047641 10.2337/diacare.27.4.874

[14] Remuzzi G, Macia M, Ruggenenti P (2006) Prevention and treatment of diabetic renal disease in type 2 diabetes: the BENEDICT study. J Am Soc Nephrol 17: S90–97. 16565256 10.1681/ASN.2005121324

[15] Brown NJ, Vaughan DE (1998) Angiotensin-converting enzyme inhibitors. Circulation 97: 1411–1420. 9577953 10.1161/01.cir.97.14.1411

[16] American Diabetes Association (2013) Standards of medical care in diabetes—2013. Diabetes Care 36 Suppl 1: S11–66. 23264422 10.2337/dc13-S011 PMC3537269

[17] Wheeler DC, Becker GJ (2013) Summary of KDIGO guideline. What do we really know about management of blood pressure in patients with chronic kidney disease? Kidney Int 83: 377–383. 10.1038/ki.2012.425 23325075

[18] Ji L, Hu D, Pan C, Weng J, Huo Y, et al (2013) Primacy of the 3B approach to control risk factors for cardiovascular disease in type 2 diabetes patients. Am J Med 126: 925 e911–922. 10.1016/j.amjmed.2013.02.035 23810406

[19] Alberti KG, Zimmet PZ (1998) Definition, diagnosis and classification of diabetes mellitus and its complications. Part 1: diagnosis and classification of diabetes mellitus provisional report of a WHO consultation. Diabet Med 15: 539–553. 9686693 10.1002/(SICI)1096-9136(199807)15:7<539::AID-DIA668>3.0.CO;2-S

[20] Levey AS, Coresh J, Greene T, Stevens LA, Zhang YL, et al (2006) Using standardized serum creatinine values in the modification of diet in renal disease study equation for estimating glomerular filtration rate. Ann Intern Med 145: 247–254. 16908915 10.7326/0003-4819-145-4-200608150-00004

[21] Heart Outcomes Prevention Evaluation (HOPE) Study Investigators (2000) Effects of ramipril on cardiovascular and microvascular outcomes in people with diabetes mellitus: results of the HOPE study and MICRO-HOPE substudy. Lancet 355: 253–259. 10675071

[22] Coyle D, Rodby R, Soroka S, Levin A, Muirhead N, et al (2007) Cost-effectiveness of irbesartan 300 mg given early versus late in patients with hypertension and a history of type 2 diabetes and renal disease: a Canadian perspective. Clin Ther 29: 1508–1523. 17825702 10.1016/j.clinthera.2007.07.029

[23] Palmer AJ, Valentine WJ, Chen R, Mehin N, Gabriel S, et al (2008) A health economic analysis of screening and optimal treatment of nephropathy in patients with type 2 diabetes and hypertension in the USA. Nephrol Dial Transplant 23: 1216–1223. 10.1093/ndt/gfn082 18359872

[24] Yang Y, Thumula V, Pace PF, Banahan BF 3rd, Wilkin NE, et al (2010) High-risk diabetic patients in Medicare Part D programs: are they getting the recommended ACEI/ARB therapy? J Gen Intern Med 25: 298–304. 10.1007/s11606-009-1242-z 20108127 PMC2842542

[25] Rosen AB (2006) Indications for and utilization of ACE inhibitors in older individuals with diabetes. Findings from the National Health and Nutrition Examination Survey 1999 to 2002. J Gen Intern Med 21: 315–319. 16686805 10.1111/j.1525-1497.2006.00351.x PMC1484715

[26] Yeh HL, Huang LY, Su S, Yang MC, Wang TC (2011) Underuse of ACE inhibitors and angiotensin II receptor blockers among patients with diabetic nephropathy in Taiwan. Health Policy 100: 196–202. 10.1016/j.healthpol.2010.11.010 21146895

[27] Rosen AB, Karter AJ, Liu JY, Selby JV, Schneider EC (2004) Use of angiotensin-converting enzyme inhibitors and angiotensin receptor blockers in high-risk clinical and ethnic groups with diabetes. J Gen Intern Med 19: 669–675. 15209606 10.1111/j.1525-1497.2004.30264.x PMC1492381

[28] Winkelmayer WC, Fischer MA, Schneeweiss S, Wang PS, Levin R, et al (2005) Underuse of ACE inhibitors and angiotensin II receptor blockers in elderly patients with diabetes. Am J Kidney Dis 46: 1080–1087. 16310574 10.1053/j.ajkd.2005.08.018

[29] Chinese Diabetes Society (2011) China Guideline for Type 2 Diabetes (2010). Beijing University Medical Press 15 p.

[30] Expert Panel on Detection, Evaluation, and Treatment of High Blood Cholesterol in Adults (2001) Executive Summary of The Third Report of The National Cholesterol Education Program (NCEP) Expert Panel on Detection, Evaluation, And Treatment of High Blood Cholesterol In Adults (Adult Treatment Panel III). JAMA 285: 2486–2497. 11368702 10.1001/jama.285.19.2486

[31] Heart Protection Study Collaborative Group (2002) MRC/BHF Heart Protection Study of cholesterol lowering with simvastatin in 20,536 high-risk individuals: a randomised placebo-controlled trial. Lancet 360: 7–22. 22115874 10.1016/S0140-6736(11)61125-2 PMC3242163

